# Single-cell transcriptomic analysis reveals tumor-immune determinants of lymph node colonization and progression in thyroid cancer

**DOI:** 10.1126/sciadv.aea4727

**Published:** 2026-07-03

**Authors:** Anthony T. Nguyen, Jolene Viramontes, Isaiah Vazquez, Catriona McWilliam, Vaishnavi Devarakonda, Regina Henson, Wendy L. Sacks, Jon Mallen-St Clair, Yufei Chen, Evan Walgama, Kevin S. Scher, Justin Moyers, Howard M. Sandler, Julie K. Jang, Zachary S. Zumsteg, Wonwoo Shon, Stephen L. Shiao, Allen S. Ho

**Affiliations:** ^1^ Department of Radiation Oncology, Cedars-Sinai Medical Center, Los Angeles, CA, USA.; ^2^ Samuel Oschin Comprehensive Cancer Institute, Cedars-Sinai Medical Center, Los Angeles, CA, USA.; ^3^ Department of Biomedical Sciences, Cedars-Sinai Medical Center, Los Angeles, CA, USA.; ^4^ Division of Endocrinology, Department of Medicine, Cedars-Sinai Medical Center, Los Angeles, CA, USA.; ^5^ Division of Otolaryngology-Head and Neck Surgery, Department of Surgery, Cedars-Sinai Medical Center, Los Angeles, CA, USA.; ^6^ Department of Surgery, Cedars-Sinai Medical Center, Los Angeles, CA, USA.; ^7^ Division of Medical Oncology, Department of Medicine, Cedars-Sinai Medical Center, Los Angeles, CA, USA.; ^8^ Department of Pathology and Laboratory Medicine, Cedars-Sinai Medical Center, Los Angeles, CA, USA.

## Abstract

Lymph node (LN) metastases are a major driver of mortality across solid cancers, including thyroid carcinomas, which are known for high rates of nodal colonization. To elucidate the determinants of nodal spread, we isolated tumor-infiltrating leukocytes from primary thyroid tumors and matched metastatic LNs for single-cell RNA sequencing with validation by multiplex immunohistochemistry. Comparing the microenvironmental alterations between primary tumors and their LNs, we found that thyrocytes and tumor-associated macrophages down-regulate the expression of multiple inflammatory cytokine receptors, including *TNFRSF12A* and *CX3CR1*, upon LN colonization. LNs were associated with the induction of regulatory T cells to suppress T cell–mediated cytotoxicity compared to matched primary tumors. Notably, tumor-infiltrating lymphocytes within LNs demonstrated increased expression of activation markers, including interleukin-7 receptor (*IL7R*). High LN expression of *IL7R* was significantly correlated with improved outcomes and can serve as a biomarker in this heterogeneous disease. Our findings on the dynamic equilibrium within LN metastases may offer conserved mechanisms for nodal colonization across solid tumors.

## Teaser

Thyroid cancers efficiently colonize lymph nodes by reprogramming the tumor microenvironment as seen at single-cell resolution.

## INTRODUCTION

Lymph node (LN) metastases are a predominant driver of mortality across solid cancers (*1*); however, there exists a wide spectrum of nodal metastatic potential between different tumor types. In particular, thyroid carcinomas demonstrate an exceptional tendency to colonize regional LNs, which necessitates extensive nodal dissections at surgery and adjuvant therapies such as radioactive iodine (RAI) to eradicate micrometastatic disease. Despite these aggressive measures, there remains a subset of patients with node-positive thyroid carcinoma who recur. Given that the incidence of thyroid malignancies has nearly tripled over the past three decades and is now the most common malignancy in adolescents and young adults (*2*, *3*), it is critical to understand how these tumors efficiently migrate from primary sites to regional tissues and how this influences prognosis.

Papillary thyroid carcinomas (PTCs) often encompass a wide spectrum of outcomes from highly indolent disease to aggressive, high-risk disease with over 20% risk of recurrence (*4*, *5*). Histologic subtypes also confer varying degrees of risk; for example, classical PTC is the most common differentiated form of thyroid cancer with relatively slow progression (*6*). Meanwhile, diffuse sclerosing PTC is associated with high rates of nodal spread, while tall cell variant of PTC represents invasive disease with greater rates of recurrence and distant metastasis (*7*, *8*). Lastly, anaplastic thyroid cancer is an undifferentiated variant with rapid progression, highly aggressive behavior, and high mortality (*9*). PTCs present with clinical nodal metastases in up to 50% of cases, and it is estimated that the frequency of occult nodal metastases may approach 90% of cases (*10*–*13*) with increasing nodal burden associated with worse oncologic outcomes (*14*). To date, large-scale bulk sequencing efforts have uncovered numerous mutually exclusive mutations within PTC, including BRAF V600E, RAS, and chromosomal translocations such as RET, NTRK, and ALK that may confer prognostic significance (*15*, *16*). Despite these advances, this approach consolidates the expression of diverse cell types and may blunt crucial distinctions between tumor components, which are accessible by single-cell techniques (*17*, *18*). Thus, there is a need to leverage high-dimensional tumor profiling at single-cell resolution to identify new biomarkers for disease progression in thyroid carcinomas as a model of nodal-centric disease.

Here, we integrate single-cell RNA sequencing (scRNA-seq) and functional analysis in thyroid carcinomas to identify immune hallmarks that facilitate LN metastasis and disease progression. By comparing the local immune repertoire between paired primary tumors and metastatic LNs, we identify multiple mechanisms that promote immune escape in PTC. We further show by functional genomics that tumor-infiltrating lymphocytes (TILs) play a critical role in maintaining immune homeostasis within nodal metastases. Our findings on the microenvironmental alterations between primary tumor and metastatic LNs demonstrate therapeutic implications for treatment de-intensification in this heterogeneous disease.

## RESULTS

### scRNA-seq of paired thyroid tumors and metastatic LNs

**Fig. 1.**
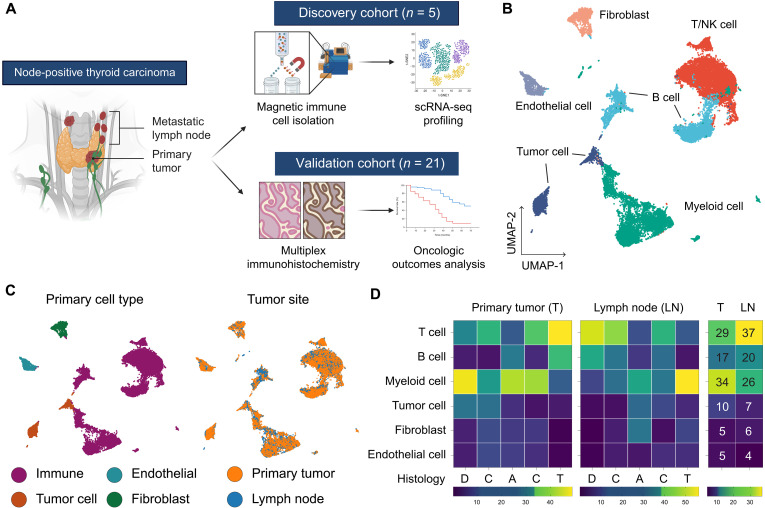
Dissecting the immune microenvironment of primary thyroid tumors and metastatic lymph nodes by scRNA-seq. (**A**) Schematic showing the experimental design. CD45^+^ tumor-infiltrating leukocytes were enriched from the paired primary tumor and metastatic LNs of patients with thyroid carcinoma for scRNA-seq at the time of surgery. Relevant immune targets were validated in a separate validation cohort of patients with PTC by mIHC with paired analysis of oncologic outcomes. (**B**) Uniform manifold approximation and projection (UMAP) plot of 31,026 cells and 9700 genes from the primary tumor and LNs labeled with major primary cell subtypes as identified by unsupervised clustering and verified by manual inspection of cell markers. (**C**) UMAP plots of primary cell types (left) or distribution of cells between primary tumor and metastatic LN (right). (**D**) Heatmap of the percentage of the six major cell types across individual patient samples from the primary tumor (left) and metastatic LN (middle). Columns denote individual patient samples from the discovery cohort. Heatmap of the average major cell type distribution (right). (A) Created in BioRender. Nguyen, A. (2026) https://BioRender.com/1fuhhm8. D, diffuse-sclerosing; C, classical; A, anaplastic; T, tall-cell variant.

To understand the differences in the local microenvironment that promote nodal metastases, we collected paired primary tumors and metastatic LNs from five patients with node-positive thyroid carcinomas to perform scRNA-seq of the local tumor microenvironment (TME). This discovery cohort of five patients all underwent total thyroidectomy and modified radical neck dissection for institutional pathologic review. We included multiple thyroid histologies within the discovery cohort, including classical PTC (*n* = 2), tall-cell variant (*n* = 1), diffuse sclerosing PTC (*n* = 1), and *BRAF*-mutated anaplastic thyroid carcinoma (*n* = 1). We reasoned that the broad inclusion of various thyroid carcinomas would allow us to capture the spectrum of microenvironmental alterations across thyroid malignancies. To validate our findings, we analyzed selected immune targets in a separate validation cohort of 21 patients with node-positive classical PTC by multiplex immunohistochemistry (mIHC) and correlation with oncologic outcomes (Fig. 1A). Clinicopathologic information and oncologic outcomes for the discovery and validation cohorts are available in table S1 and fig. S1.

We reasoned that there are both tumor- and immune-driven factors that generate a permissive TME for nodal colonization. Since immune cells are highly underrepresented in both primary tumors and metastatic LNs, we enriched for CD45^+^ tumor-infiltrating leukocytes before generating scRNA-seq libraries for analysis (Fig. 1A). A total of 31,026 cells expressing 9700 genes were analyzed by unsupervised clustering, which identified six main cell populations (Fig. 1, B and C, and fig. S2). CD45^+^ tumor-infiltrating leukocytes were the dominant captured cell type, representing ~80% of all cells, including myeloid cells, T/natural killer (NK) cells, and B/plasma cells. In addition, we captured a small subset of nonimmune cells, including thyroid carcinoma cells, endothelial cells, and fibroblasts. We observed each of these six cell populations from both primary tumors and metastatic LNs at similar frequencies across each patient sample (Fig. 1D). In general, T/NK cells were identified more frequently from LN samples versus primary tumors. By contrast, myeloid cells were more commonly sequenced from primary tumor specimens, suggesting potential differences in the immune composition of these two sites of disease (Fig. 1D).

### Immune escape by thyroid carcinoma cells in metastatic LNs

**Fig. 2.**
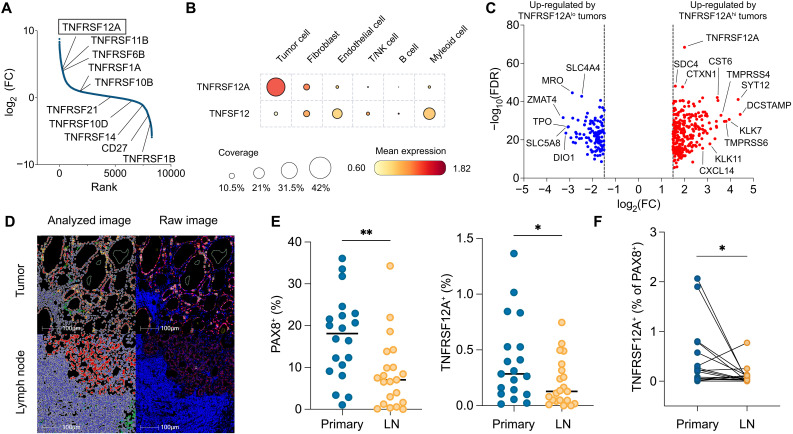
Thyroid tumor cells down-regulate *TNFRSF12A* expression upon metastatic LN colonization. (**A**) Thyroid tumor cell ranked log_2_-transformed fold change (FC) in gene expression between tumor cells and other nontumor cells. TNF receptor superfamily genes are labeled. (**B**) Bubble heatmap showing expression of *TNFRSF12A* and its ligand *TNFSF12* in the single-cell dataset. *n* = 5 pooled patients. (**C**) Volcano plot of differentially expressed genes between *TNFRSF12A*-high and *TNFRSF12A*-low thyroid cancers from TCGA, using a cutoff of log_2_FC >1.5 or <−1.5. (**D**) Sample mIHC staining of primary tumor (upper panels) and metastatic LN (lower panels) for PAX8 (red) and TNFRSF12A (yellow). Left, image quantification by HALO; right, raw image of immunohistochemistry staining. Scale bar, 100 μm. (**E**) Percentage of positive cells per total identified nuclei by immunohistochemistry staining for PAX8 (left) and TNFRSF12A (right). The horizontal line indicates the mean. Each dot represents an individual patient sample. (*n* = 20 for primary, and *n* = 21 for LN, left). (*n* = 19 for primary, and *n* = 21 for LN, right). *P* = 0.004, left. *P* = 0.047, right. (**F**) Pairwise analysis of primary tumor and metastatic LN for the percentage of PAX8-positive cells that express TNFRSF12A. (*n* = 17 pairs). *P* = 0.0498. *P* values were determined by unpaired two-sided Student’s *t* test in (E), and by paired two-sided Student’s *t* test in (F). *P < 0.05 and **P < 0.01.

Given that the immune microenvironment is shaped by the local tumor cells, we first examined whether there were any differences between the thyroid carcinoma cells in the primary tumor and metastatic LN. We analyzed the 2681 thyroid carcinoma cells that were captured by scRNA-seq in our discovery cohort to identify any potential tumor-intrinsic factors that may facilitate nodal colonization and promote immune evasion. Thyroid carcinoma cells are highly sensitive to inflammatory signaling through tumor necrosis factor–α (TNFα), which can induce tumor cell apoptosis (*19*, *20*). Patients with thyroid carcinomas that express high levels of the TNFα receptor (*TNFRSF1A*) have improved overall survival in an analysis of The Cancer Genome Atlas (TCGA), as these tumors are more susceptible to apoptosis-inducing inflammatory signaling (fig. S3A). We hypothesized that modulation of TNF signaling, which can induce tumor cell apoptosis, is associated with improved survival and may serve as an evolutionary method for thyroid carcinoma cells to colonize distant sites. To this end, we queried the expression of the TNF receptor superfamily members, comparing expression between thyroid tumor cells and all other cells by scRNA-seq of our discovery cohort (Fig. 2A). Thyroid tumor cells in both primary tumors and metastatic LNs expressed multiple TNF receptor superfamily genes in high abundance compared to other stromal and immune cells. Of the enriched TNF receptor superfamily genes by thyroid tumor cells, *TNFRSF1A* and *TNFRSF12A* were significantly associated with overall survival in thyroid carcinoma (fig. S3, B and C). Only *TNFRSF12A* remained associated with survival in both node-negative and node-positive disease (fig. S3D). We detected the expression of *TNFRSF12A* primarily in tumor cells and the expression of its ligand *TNFSF12* predominantly in myeloid cells and endothelial cells in both primary tumor and metastatic LN (Fig. 2B).

To elucidate the role of *TNFRSF12A* on metastatic potential, we compared the global gene expression of *TNFRSF12A*-high and *TNFRSF12A*-low thyroid cancers from TCGA. We found that *TNFRSF12A*-high tumors up-regulated several genes and pathways implicated in extracellular remodeling, invasion, and cytokine-driven inflammation (Fig. 2C and fig. S4). To reconcile the unexpected association of *TNFRSF12A* with both improved survival and pathways of tumor progression, we compared the expression of *TNFRSF12A* between primary tumors and metastatic LNs in our validation cohort of patients with PTC by mIHC (Fig. 2, D to F). Metastatic LNs had fewer PAX8^+^ thyroid carcinoma cells, which expressed significantly less *TNFRSF12A* compared to primary tumor cells (Fig. 2E). To confirm this finding, we analyzed all available pairwise samples for PAX8^+^TNFRSF12A^+^ tumor cells, which showed a significant reduction in TNFRSF12A-expressing thyroid carcinoma cells in metastatic LN samples (Fig. 2F). On the basis of these observations, we hypothesize that thyroid tumor cells may down-regulate the expression of *TNFRSF12A*, among other TNF receptor superfamily members, to avoid apoptosis by TNF signaling at distant sites.

### Innate immune dysfunction and outcomes in thyroid carcinoma

**Fig. 3.**
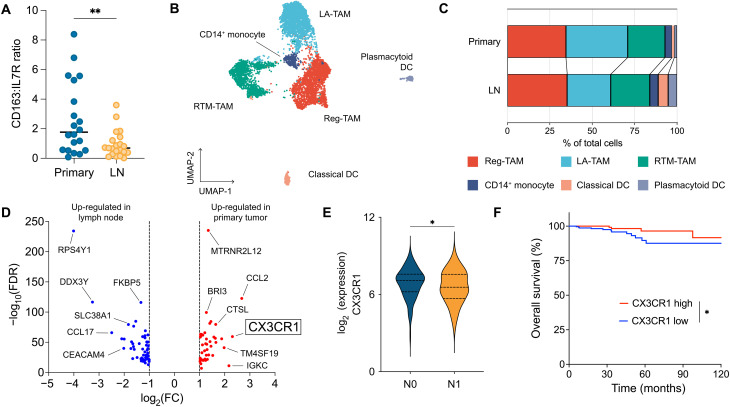
*CX3CR1*-expressing TAMs are enriched in primary tumors and are associated with survival. (**A**) Ratio of CD163-positive cells to IL7R-positive cells by immunohistochemistry staining, stratified by primary tumor versus metastatic LN. The horizontal bar indicates the mean value. (*n* = 20 for primary, and *n* = 21 for LN). *P* = 0.006. (**B**) UMAP plot showing reclustered innate immune cells, including six innate immune cell subtypes identified by manual inspection of known functional markers. (**C**) Percentage distribution of innate immune cell subtypes, including TAMs and DCs, between the primary tumor and metastatic LN. (**D**) Volcano plot of differentially expressed genes of innate immune cells between primary tumor and metastatic LN, using a cutoff of log_2_FC >1 or <−1. (**E**) Violin plot of *CX3CR1* mRNA expression from patients with node-negative thyroid carcinoma (N0) versus node-positive thyroid carcinoma (N1). *P* = 0.014. (**F**) Kaplan-Meier estimate of overall survival in patients with thyroid carcinoma stratified by median mRNA expression of *CX3CR1*. *P* = 0.043. *P* values were determined by unpaired two-sided Student’s *t* test in (A) and (E), and by log-rank test in (**F**). *P < 0.05 and **P < 0.01. LA-TAM, lipid-associated TAM; RTM-TAM, resident-tissue macrophage-like TAM; Reg-TAM, immune regulatory TAM; FDR, false discovery rate.

We then sought to identify differences in the immune microenvironment between primary thyroid carcinomas and metastatic LNs. To begin, we focused on the innate immune response, including tumor-associated macrophages (TAMs) and dendritic cells (DCs). Consistent with scRNA-seq analysis of our discovery cohort, we observed an increase in CD163^+^ TAMs as a percentage of the immune compartment within primary tumors compared to nodal metastases by mIHC in our PTC validation cohort (Fig. 3A). We reasoned that there are differences in the functional capacity of innate immune cells in addition to their increased recruitment to primary tumors. To this end, we performed a subcluster analysis of the scRNA-seq data from the discovery cohort and identified six myeloid cell clusters, including lipid-associated TAMs (LA-TAMs), immune regulatory TAMs (Reg-TAMS), resident-tissue macrophage-like TAMs (RTM-TAMs), CD14^+^CD16^low^ monocytes, classical DCs, and plasmacytoid DCs (Fig. 3B and fig. S5, A to E). Comparing the distribution of these myeloid subsets between disease sites, we observed increased LA-TAMs within the primary tumor, which may suggest increased phagocytosis and lipid-processing due to increased tumor bulk at the primary site. By contrast, we observed an enrichment in classical and plasmacytoid DCs with metastatic LNs (Fig. 3C).

To identify specific molecular targets within these tumor-associated myeloid cells, we conducted differential gene expression analysis on all myeloid cells from the primary tumor and metastatic LN (Fig. 3D). We found that myeloid cells within the primary tumor demonstrated increased expression of multiple genes, including *CX3CR1*, which is an inflammatory chemokine receptor that can mediate leukocyte chemotaxis into the local TME. *CCL2* was also significantly up-regulated by myeloid cells within the primary tumor; however, *CCL2* is also expressed by fibroblasts and endothelial cells in our dataset. Given that myeloid cells, and specifically LA-TAMs, are enriched in the primary tumor and respond to inflammatory cues, we reasoned that these cells may be actively suppressing tumors within the primary site. We observed increased expression of *CX3CR1* in localized thyroid carcinomas without LN metastases (N0) from TCGA (Fig. 3E). Moreover, we found that higher expression of *CX3CR1* and *APOC1*, a canonical marker of LA-TAMs, is positively correlated with overall survival in patients with thyroid carcinoma from TCGA (Fig. 3F and fig. S6). Thus, compared to metastatic LNs, primary tumors were associated with increased macrophages and LA-TAMs. Moreover, down-regulation of these pathways may be associated with locally advanced disease and subpar oncologic outcomes.

### Regulatory T cells promote immunosuppression within metastatic LNs

**Fig. 4.**
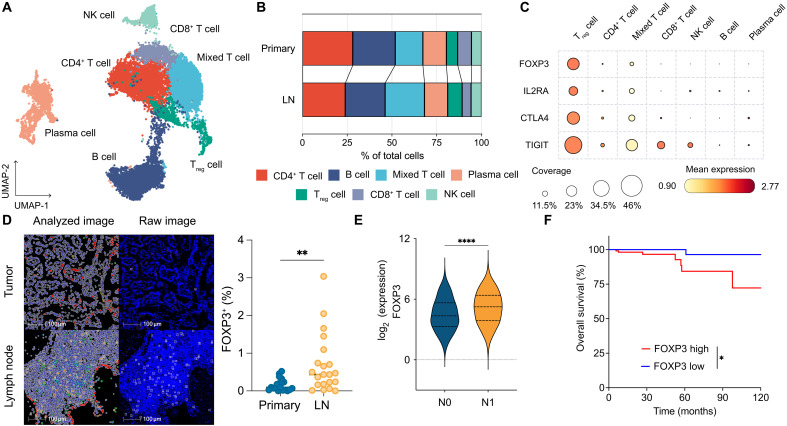
Regulatory T cells promote an immunosuppressive microenvironment within metastatic LNs. (**A**) UMAP plot of reclustered TILs with six annotated cell subtypes based on functional markers. (**B**) Percentage distribution of TIL subtypes, including T, B, and NK cells, between the primary tumor and metastatic LN. (**C**) Bubble heatmap showing the expression of canonical T_reg_ markers as well as markers of exhaustion. *n* = 5 pooled patients. (**D**) Representative immunohistochemistry staining of FOXP3 (orange) between the primary tumor (top row) and metastatic LN (bottom row). Right column, raw image of immunohistochemistry staining; left column, quantified images as analyzed by HALO. Percentage of FOXP3-positive cells per total identified nuclei stratified by primary tumor versus metastatic LN (right). Mean value is indicated by the horizontal line. Each dot is an individual patient sample. (*n* = 19 for primary, and *n* = 21 for LN). *P* = 0.006 (**E**) Violin plot of *FOXP3* mRNA expression between patients with node-negative thyroid carcinoma (N0) and node-positive thyroid carcinoma (N1). *P* < 0.0001. (**F**) Kaplan-Meier plot of overall survival in patients with node-positive thyroid carcinoma stratified by median mRNA expression of *FOXP3*. *P* = 0.018. *P* values were determined by unpaired two-sided Student’s *t* test in (D) and (E), and by log-rank test in (F). *P < 0.05, **P < 0.01, and ****P < 0.0001. T_reg_, regulatory T cell.

Next, we explored the differences in the adaptive immune response between primary thyroid carcinomas and metastatic LNs. To this end, we performed a subcluster analysis of 16,760 TILs captured by scRNA-seq in our discovery cohort, including B cells, T cells, NK cells, and plasma cells (Fig. 4A and fig. S7). We identified six TIL subtypes by unsupervised clustering, which encompassed B cells, plasma cells, NK cells, CD4^+^ T cells, CD8^+^ T cells, mixed T cells, and regulatory T cells (T_reg_). The composition of TIL subtypes varied between the primary tumor and metastatic LN (Fig. 4B). To identify immune-specific factors that promote LN colonization, we focused on cell subtypes that were enriched within nodal metastases, which included T_reg_ cells and mixed T cells. Given that the T_reg_ cluster expressed *FOXP3* among other canonical markers and markers of exhaustion (Fig. 4C), we then compared the expression of *FOXP3* between primary tumor and metastatic LN within our validation cohort by mIHC. Consistent with scRNA-seq analysis, we observed an increase in FOXP3 staining in metastatic LNs compared to primary tumors (Fig. 4D). As T_reg_ cells have potent immunosuppressive capacity, these data suggest that thyroid carcinomas can license T_reg_ cells to avoid immune recognition and to allow for colonization of distant LNs. Although we observed fewer T_reg_ cells within the primary tumor, we reasoned that T_reg_ cells within the primary site may also influence local control and long-term oncologic outcomes by suppressing local immune responses. Consistent with this hypothesis, expression of *FOXP3* was significantly higher in node-positive thyroid carcinomas compared to localized, node-negative disease in patients with thyroid carcinoma from TCGA (Fig. 4E). Moreover, among patients with node-positive thyroid cancer in TCGA, elevated *FOXP3* expression was associated with inferior survival outcomes (Fig. 4F). Put together, these data indicate that T_reg_ cells may facilitate LN metastases through the local nodal microenvironment and by suppressing immune responses within the primary tumor.

### Immune activation via IL7R is prognostic of outcomes in thyroid carcinoma

**Fig. 5.**
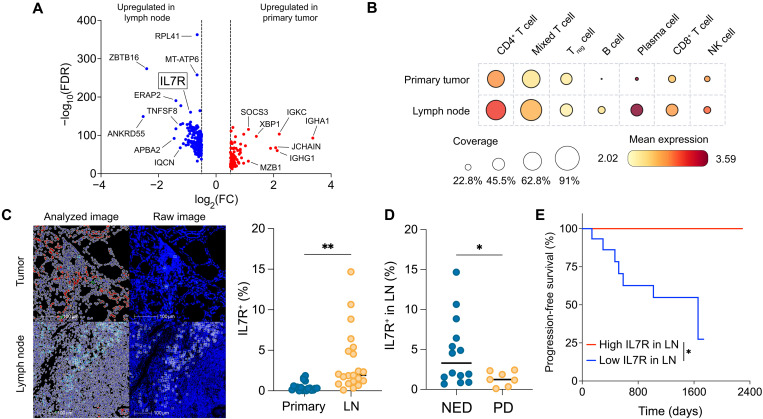
IL7R is associated with enhanced immune activation in metastatic LNs and improved progression-free survival. (**A**) Volcano plot of differentially expressed genes in TILs between the primary tumor (red) and metastatic LNs (blue). A cutoff of log_2_(FC) >0.5 or <−0.5 was used to identify target genes. (**B**) Bubble heatmap showing expression of *IL7R* in different TIL subsets between primary tumor and LN. *n* = 5 pooled patients. (**C**) Representative immunohistochemistry staining of IL7R (cyan) between the primary tumor (top row) and metastatic LN (bottom row). Right column, raw image of immunohistochemistry staining; left column, quantified images as analyzed by HALO. Percentage of IL7R-positive cells per total identified nuclei by immunohistochemistry staining. The horizontal line indicates the mean value. Each dot represents an individual patient. (*n* = 19 for primary, and *n* = 21 for LN). *P* = 0.001. (**D**) Percentage of IL7R-positive cells within metastatic LNs in the validation cohort, stratified by patients with NED or with progression. Individual patients are represented by each circle. (*n* = 14 for NED, and *n* = 7 for PD). *P* = 0.014. (**E**) Kaplan-Meier estimate for progression-free survival in the validation cohort as stratified by high or low IL7R staining within the metastatic LN. *P* = 0.030. *P* values were determined by unpaired two-sided Student’s *t* test with Welch’s correction in (C) and (D), and by log-rank test in (E). *P < 0.05 and **P < 0.01.

Although T_reg_ cells may facilitate nodal colonization, we reasoned that there are counteracting immune drivers that prevent disease progression and distant metastases. To this end, we performed differential gene expression comparing TILs between primary tumors and metastatic LNs in our discovery cohort (Fig. 5A). We identified a host of differentially expressed genes, suggesting two disparate TIL states between primary and metastatic sites. In particular, one of the most up-regulated genes within nodal TILs was the interleukin-7 receptor (*IL7R*; also known as CD127). *IL-7* signaling through *IL7R* plays a critical role in the homeostasis of CD4^+^ memory T cells by promoting their proliferation, differentiation, and survival. Consistent with its described function, we observed *IL7R* expression on both CD4^+^ T cells and mixed T cells in our scRNA-seq dataset (Fig. 5B). We then performed mIHC in our validation cohort to confirm that *IL7R* expression is up-regulated in metastatic LNs. We observed increased *IL7R*-expressing cells within nodal metastases versus primary tumors in our validation cohort (Fig. 5C).

Given that IL7R is associated with survival in other solid cancers, including melanoma, we evaluated the prognostic role of *IL7R* expression in thyroid carcinoma. We hypothesized that *IL7R* expression specifically within the LN and not in the primary tumor is prognostic of outcomes. Within our validation cohort, we compared the levels of *IL7R* staining within nodal metastasis between patients with no evidence of disease (NED) or with documented progression or death. This analysis revealed that there were significantly higher levels of nodal *IL7R* expression among patients who remained NED despite high-risk, node-positive disease versus patients with disease progression (*P* = 0.014, Fig. 5D). Progression-free survival was significantly extended in patients with the highest quartile of *IL7R* expression compared to all remaining patients (log-rank *P* = 0.030, Fig. 5E). Patients with the highest quartile of *IL7R* expression within LNs demonstrated a 100% progression-free survival, highlighting the prognostic potential of this biomarker. By contrast, *IL7R* expression within the primary tumor or other immune markers within the LN were not prognostic of outcomes in this cohort (fig. S8).

## DISCUSSION

Here, we defined the microenvironmental alterations that allow thyroid carcinomas to efficiently colonize distant metastatic LNs by high-dimensional tumor profiling at single-cell resolution. Our evaluation of the TME of thyroid carcinomas revealed multiple tumor-intrinsic and tumor-extrinsic signatures that are prognostic for outcomes in a large population cohort. Further, our studies unexpectedly revealed *Il7R* as a nodal-specific biomarker predictive of recurrence that may better stratify patients with LN metastases. Prospective studies will be required to validate this biomarker to identify candidates for treatment calibration.

Recently, scRNA-seq has provided valuable insight into thyroid carcinomas and thyroid-related diseases with a focus on cancer initiation, progression, and anaplastic transformation (*18*, *21*, *22*). Here, we leverage functional genomics by scRNA-seq to advance our understanding of nodal colonization within thyroid cancers. First, we sought to identify tumor-intrinsic differences between primary tumors and tumor-draining LNs. We found that thyrocytes significantly down-regulate the expression of the TNF receptor superfamily member, *TNFRSF12A*, within nodal metastases compared to primary tumors. TNFRSF12A serves as the cell-surface receptor for TNFSF12 and plays a pivotal role in normal tissue homeostasis and repair. It appears to have pleiotropic properties, harboring antitumor properties in certain cancer types and contexts. Outside of early phase 1 studies (*23*), little has been explored regarding its use as a therapeutic target. Since TNF signaling can induce apoptosis in thyroid carcinoma cells (*19*, *20*, *24*), loss of *TNFRSF12A* upon migration to LNs may desensitize PTCs to locally produced TNF cytokines within the nodal microenvironment.

We then focused on tumor-extrinsic differences between matched primary tumors and metastatic LNs in PTC by enriching for tumor-infiltrating leukocytes. Innate immune cells, including TAMs, were more predominantly identified within the primary tumor versus metastatic LN. Several pan-cancer studies have used scRNA-seq to characterize the full diversity of TAMs to correlate with cancer outcomes (*25*–*27*). Our analysis revealed an enrichment of LA-TAMs expressing several markers of lipid metabolism within primary tumors, which may function to wall off the primary site. Further, we identified an up-regulation of the inflammatory cytokine receptor *CX3CR1* within TAMs in the primary tumor. *CX3CR1* and *APOC1*, a marker for LA-TAMs, were significantly associated with survival in thyroid cancers. CX3CR1 in particular has been implicated in cell migration, angiogenesis, and apoptosis resistance in multiple other cancers (*28*). Multiple CX3CR1 antagonists are currently under study, while active trials are investigating CX3CR1 levels as a biomarker to predict immunotherapy efficacy in lung cancers (*29*).

Our data indicate that the microenvironment within metastatic LNs exists in a fine equilibrium between immunosuppressive and immune-activating elements. Given the suppressive activity of T_regs_, it is not unexpected that high levels of *FOXP3*, a canonical T_reg_ marker, within the primary tumor are associated with locally advanced PTCs and poor long-term overall survival (*30*). However, by scRNA-seq and mIHC, we found that T_regs_ are significantly and further enriched within metastatic LNs compared to matched primary tumors. This finding suggests that the T cell landscape is vastly different between primary tumors and LNs and that PTCs may leverage the suppressive capacity of T_regs_ to colonize the hostile microenvironment of distant LNs. Further, these data are consistent with preclinical studies in melanoma demonstrating that LN metastases can induce T_regs_ and suppress T cell responses to promote distant metastases (*31*). Although T_regs_ were found in relatively small abundance in both discovery and validation cohorts, T_regs_ have been shown to exert disproportionate immunosuppressive influence in thyroid cancers and across diverse tumor types (*32*–*34*).

We then compared the global transcriptional landscape of TILs between disease sites and identified an up-regulation of *IL7R* at the transcript and protein level within tumor-draining LNs. Given that *IL7R* is essential for T cell development, survival, and homeostasis and is associated with improved outcomes in other solid tumors (*35*, *36*), we found that expression of *IL7R* within metastatic LNs is highly prognostic of oncologic outcomes within a cohort of patients with locally advanced PTC. Patients within the highest quartile of nodal *IL7R* expression demonstrated a 100% progression-free survival. This finding is consistent with the observation that tumor-specific T cells within tumor-draining LNs express high levels of *IL7R* (*36*). Thus, T cell activation and homeostasis via *IL7R* may counterbalance the immunosuppressive capacity of T_regs_ within the TME of tumor-draining LNs.

Given their role in antitumor immunity, IL-7 has increasingly been explored in trials to remodel the TME and enhance the effect of immunotherapy. Recombinant, long-acting IL-7 has now undergone clinical testing alone or in combination with other immunotherapies to stimulate T cell antitumor function in more than a dozen phase 1-2 studies encompassing glioblastoma, bladder carcinoma, lymphoma, and head and neck cancers (*37*). This has also expanded to studies in the refractory setting (*38*). Moreover, IL-7 has been exploited as an “immune reconstitution” mediator to replenish T cells in immunodepleted cancer patients (*39*). Such studies demonstrate clinical ability for IL-7 to potentiate antitumor immunity via activation of TILs and restore tumor susceptibility to partner drug therapy. Together, the breadth of trials investigating IL-7 strongly suggests its vast potential as a viable treatment approach across tumors.

Notably, IL-7 appears to elicit context-dependent effects, with some investigators advocating a paradoxical role in promoting regional and distant metastasis. Preclinical studies in non–small cell lung cancer models have demonstrated IL-7 expression in bone metastases, supporting work that demonstrates its ability to promote osteoclast activation (*40*). Other studies have further identified an association of IL-7 with nodal spread, distant spread, and poor prognosis in prostate, breast, and colorectal cancer (*41*–*43*), potentially through AKT/NFKB up-regulation and MMP expression. The pleotropic character of IL-7 has remained challenging to clarify due to cross-talk between competing factors that include cancer histology and TME, in a time- or dose-dependent manner distinct from its antitumor immunity role.

Nodal metastases are a major driver of mortality across solid cancers, including PTCs (*1*, *14*). However, there is a wide spectrum of outcomes in node-positive patients with PTC, ranging from indolent disease to rapid recurrence, with initial molecular strategies yielding mixed success (*44*, *45*). Identification of bad actors will optimize treatment strategies while providing evidence-based reassurance. RAI is commonly administered for more advanced cases at higher risk of recurrence or mortality, but significant controversy exists regarding (i) indications and (ii) dosage, given its adverse side effect profile. Evolving national guidance recommends consideration of RAI for >5 metastatic nodes or nodal size >3 cm, but this is a crude measure in need of further refinement, to avoid overtreating patients and causing needless toxicity for marginal gains in survival. Thus, there is a clinically unmet need to better identify patients at high risk for disease progression. Large population-based databases, including TCGA, provide valuable data on the genomic landscape of PTCs with clinicopathologic correlations (*15*); however, transcriptional data from these studies are often derived from the primary tumor with little to no data about the transcriptional state of nodal metastases. Our study is the first, to our knowledge, to leverage single-cell transcriptomics to identify a nodal-specific biomarker that is prognostic of outcomes in PTC. On the basis of our data, evaluating the expression of *IL7R* can potentially be used to identify patients with more aggressive disease who are candidates for treatment intensification. Given that the immune landscape is vastly different between primary tumors and tumor-draining LNs, it would make sense to interrogate the local microenvironment of the distant LN to better prognosticate outcomes in PTC. These data warrant the investigation of *IL7R* as a prognostic biomarker in larger, prospective cohorts as well as the predictive capacity for *IL7R* to drive treatment de-intensification.

There are several limitations to this study. First, we included a heterogeneous group of patients for our discovery cohort, including classical PTC, tall-cell variant, diffuse sclerosing PTC, and *BRAF*-mutated anaplastic thyroid carcinoma. We reasoned that we wanted to capture the full spectrum of thyroid carcinomas by scRNA-seq; however, the inclusion of multiple disease variants may skew the distribution of immune cells and alter downstream functional genomics analyses. Further, the heterogeneity of subtypes and the limited number of well-differentiated thyroid carcinomas precluded a comprehensive analysis of tumor-intrinsic mechanisms of nodal colonization, which were mitigated by focusing on immune cell niches in metastasis. Another limitation of this study is the size of our validation cohort for mIHC analysis. Although we validated our scRNA-seq targets within a limited cohort, we recognize that our findings require additional prospective validation in a larger patient cohort. Although patients with node-negative (N0) PTC were excluded from the discovery and validation cohorts, these patients may have additional microenvironmental alterations that may promote micrometastases. Last, we were limited by the number of markers from scRNA-seq that we could validate with mIHC. For example, T cell activation is associated with tertiary lymphoid structure (TLS) development, which underlies the response to immunotherapy (*46*, *47*). However, we did not identify B cells in abundance by scRNA-seq and did not assess TLS formation in our validation cohort.

In summary, we leveraged single-cell transcriptomics to identify immune and nonimmune hallmarks that facilitate nodal colonization in thyroid carcinomas. Our study not only provides a comprehensive landscape of the microenvironmental alterations between primary tumor and metastatic LNs but also identifies a nodal-specific biomarker that is prognostic for oncologic outcomes in this highly heterogeneous disease. Our data shed additional mechanistic insight into how PTCs can colonize distant sites with implications for therapeutic interventions.

## MATERIALS AND METHODS

### Sex as a biological variable

Although thyroid carcinomas are more prevalent in women, we included both female and male patients in our discovery and validation cohorts to capture the range of microenvironmental alterations and to increase the generalizability of our results.

### Ethical statement

This study was reviewed and approved by the Institutional Review Board of Cedars-Sinai Medical Center (CSMC). Written informed consent was obtained from patients per approved protocols.

### Human specimens

Five patients who underwent total thyroidectomy and modified radical neck dissection at CSMC were included in the discovery cohort. In total, 10 fresh surgical specimens (five primary tumors and five metastatic LNs with macroscopic tumor involvement) were surgically resected and processed immediately for downstream tumor-infiltrating leukocyte purification and scRNA-seq. Each paired patient sample was analyzed by scRNA-seq separately. Twenty-one independent patients with PTC who underwent surgery at CSMC between 2018 and 2021 were included in the validation cohort. In total, 42 surgical specimens (21 primary tumors and 21 metastatic LNs) were de-identified and used for analysis. Hematoxylin and eosin–stained sections of each sample were prepared and reviewed by a dedicated head and neck cancer pathologist. In the discovery cohort, 5 μM formalin-fixed, paraffin-embedded (FFPE) tissue sections were used to confirm PTC diagnosis by the head and neck pathologist. Summary of clinicopathologic variables and survival outcomes for the discovery and validation cohorts are available in table S1 and fig. S1.

### Sample preparation

To isolate tumor-infiltrating leukocytes from tumor samples, surgical specimens were cut with a sterile scalpel into approximately 2-mm pieces and placed into a gentleMACS C-tube (Miltenyi Biotec) with 4.7-ml of ice-cold Dulbecco’s Modified Eagle Medium (DMEM). Tumors were dissociated using enzymes from the Tumor Dissociation Kit, Human (Miltenyi Biotec) per the manufacturer’s protocol for the digestion of tough tumors, including head and neck cancers. The C-tube with the enzymatic digestion was placed onto the gentleMACS Octo Dissociator with Heaters (Miltenyi Biotec) and dissociated using the 37C_h_TDK_3 program. After completion of the dissociation protocol, the sample was filtered through a 70-μm cell strainer pre-wetted with heat-inactivated fetal bovine serum (FBS), followed by washing with 5 ml of heat-inactivated FBS. The samples were centrifuged at 4°C, and the pellet was resuspended in 2 ml of ice-cold DMEM. Following cell counting by the Countess II Automated Cell Counter (Thermo Fisher Scientific), the sample was resuspended in MACS running buffer (Miltenyi Biotec) and incubated with anti-human CD45 microbeads (Miltenyi Biotec, RRID:AB_2783001). Samples were incubated at 4°C in the dark for 15 min. After washing and filtration, samples were loaded onto the autoMACS Pro Separator (Miltenyi Biotec) using the Possel_S program. During CD45 cell isolation, reagents for the Single Cell 3′ v1 Assay (10x Genomics) were thawed to room temperature for 30 min. Upon completion of the autoMACS separation, the CD45-positive fraction was resuspended in DMEM, with cell count and viability performed by the Countess II Automated Cell Counter (Thermo Fisher Scientific).

### Single-cell RNA sequencing

Following CD45-positive selection, samples were diluted to 1000 cells/μl and processed according to the Single Cell 3′ v1 assay (10x Genomics) with a targeted 10,000 cell recovery. Gene expression libraries were prepared according to the Single Cell 3′ v1 protocol (10x Genomics) and were sequenced using paired-end 150 bp reads on the Novaseq 6000 (Illumina) through the CSMC Applied Genomics, Computation & Translational Core. Demultiplexed fastq files for gene expression libraries were processed by Cellranger (version 4.0.0, GRCh38-3.0.0) transcriptome reference.

### Single-cell dimensionality reduction and clustering

Analysis was performed using BBrowserX (BioTuring) for quality control and downstream bioinformatic analysis. We filtered out cells with low-quality based on mitochondrial gene counts <25% and read counts between 10 and 1,000,000. Batch correction was performed using Harmony, and UMAP embedding and Louvain clustering were performed using the top 2000 highly variable genes after batch correction. Major cell types were annotated based on canonical lineage-defining makers: myeloid cells (*ITGAM*, *ITGAX*, *CD163*, *S100A8*, *S100A9*, *LYZ*, and *CD14*), T and NK cells (*CD3E*, *CD3D*, *CD8A*, *CD247*, *NKG7*, and *CD3G*), B and plasma cells (*CD19*, *CD79A*, *CD79B*, *IGHD*, and *IGHM*), thyroid epithelial cells (*PAX8*, *TG*, *EPCAM*, *KRT18*, and *KRT19*), endothelial cells (*PECAM1*, *AQP1*, *CALCRL*, *VWF*, *CD45*, and *CDH5*), and fibroblasts (*COL1A1*, *COL1A2*, *COL3A1*, *COL6A2*, and *ACTA2*). Higher resolution reclustering analysis was performed within each major cell type to identify additional subclusters.

### Differential gene analysis

To identify clustering-defining markers, we performed differential gene expression analysis on unsupervised clusters within each major cell type using the Venice algorithm, which is a nonparametric statistical test. Cluster-specific markers were identified as genes with a Bonferroni-adjusted *P* value [false discovery rate (FDR)] <0.05 or log_2_ fold change (FC) >0.5 or 1. Pathway and upstream regulator analysis was performed using ingenuity pathway analysis (QIAGEN) on differentially expressed genes using a cutoff of log_2_ FC >1.5 or <−1.5 and FDR <0.05.

### Antibodies

The following antibodies and concentrations were used for mIHC: mouse anti-human anti-CD127 (Thermo Fisher Scientific, catalog no. 14-1278-82, RRID:AB_657591) at 1:100; rabbit anti-human anti-FOXP3 (Cell Signaling Technology, catalog no. 98377, RRID:AB_2747370) at 1:100; mouse anti-human anti-CD163 (Thermo Fisher Scientific, catalog no. MA5-11458, RRID:AB_10982556) at 1:100; rabbit anti-human anti-CD266 (Thermo Fisher Scientific, catalog MA5-38151, RRID:AB_2898069) at 1:150; mouse anti-human anti-PAX8 (Thermo Fisher Scientific, catalog MA1-117, RRID:AB_2536828) at 1:50.

### Multiplex immunohistochemistry

FFPE tumor and LN tissues were fixed onto a slide for staining. Slides were baked at 60°C, deparaffinized with xylene, and rehydrated with ethanol washes. After the first hematoxylin stain (DAKO, S3301), antigen retrieval was performed by steam heating with 1× citrate buffer. To block for endogenous peroxidase activity, slides were incubated with Dual Endogenous Enzyme Block (DAKO, S2003) followed by blocking buffer (5% normal goat serum in 2.5% BSA). Following staining with each target primary antibody as well as two additional hematoxylin stains, tissue sections were washed in tris-buffered saline with Tween 20 (TBST) and subsequently stained with HistoFine (M/R/G) Simple Stain MAX PO (Nichirei Bioscience Inc., 414152F) secondary antibodies for 30 min. Tissue sections were washed again with TBST, then MilliQ water, and incubated with AEC substrate (Vector Labs, SK-4200) for stain development. To remove the stain, tissue sections were washed with a series of ethanol washes and incubated with a Dual Endogenous Enzyme Block. Tissues were then re-stained with the next antibody in the panel. To amplify the signal of the final antibody in the panel, an avidin-biotin complex was used with a biotinylated goat anti-mouse immunoglobulin G (Jackson ImmunoResearch, 115-065-003, RRID:AB_2338557) secondary antibody. Sections were washed with TBST and incubated with ABC-HRP (VectorLabs, PK-6100). After ABC incubation, tissue sections were washed again with TBST, then MilliQ water, and finally incubated with AEC substrate for stain development. To quench the AEC substrate, sections were submerged in MilliQ water and then TBST until imaging.

### Imaging analysis

Following staining, coverslips were applied to tissue sections with TBST, and whole-slide images were scanned at 20× and 40× magnification on the Aperio ScanScope AT Turbo slide scanner (Leica Biosystems). Full-slide images were quantified using the HALO software (Indica Labs, version 3.6) using the following modules: Deconvolution version 1.1.8 and HighPlex FL version 4.2.14. Staining was quantified as the number of positive cells divided by the total number of nuclei per section.

### TCGA analysis

For oncologic evaluation of candidate genes, we retrieved anonymized gene expression data from TCGA through the publicly available cBioPortal for Cancer Genomics (https://cbioportal.org/) using the THCA thyroid cancer cohort. Patients were divided into bins based on the median gene expression data (RNA Seq V2 RSEM) for each candidate gene, excluding overlapping samples and patients. Overall survival was compared by the log-rank test. Node-negative patients were identified based on the AJCC pathologic LN stage (AJCC_NODES_PATHOLOGIC_PN) as N0 (*n* = 231); node-positive patients were identified by the same criteria as N1a, N1b, or N1 (*n* = 226). Patients with unavailable pathologic nodal staging (NX or NA) were excluded from node-positive and node-negative comparisons.

### Human validation

We used patients with PTC from the validation cohort (*n* = 21) to validate *IL7R* as a biomarker for recurrence in thyroid carcinoma. The primary end point of the human validation was progression-free survival as determined from the time of definitive surgery to an event of local or distant progression or recurrence, or death due to any cause, whichever occurred first. LN expression of *IL7R*, quantified as percent of positively staining cells divided by the total number of nuclei, was divided by the highest quartile of expression compared to the remaining cohort. The Kaplan-Meier method was used to estimate progression-free survival, with a comparison performed by the log-rank test.

### Statistical analysis

All statistical analyses were performed using Prism (version 10.4.2). Statistical tests include unpaired and paired Student’s *t* test and unpaired Student’s *t* test with Welch’s correction. Survival curves were constructed using the Kaplan-Meier method with comparison by the log-rank test. Two-sided tests were used with significance calculated at the 0.05 level.
