# Supplementary material for: Single-cell transcriptomic analysis reveals tumor-immune determinants of lymph node colonization and progression in thyroid cancer
Source: Sci Adv. 2026 Jul 3;12(27):eaea4727. doi: 10.1126/sciadv.aea4727 (PMC13330817; doi:10.1126/sciadv.aea4727)
Supplement: Supplementary file 1 — Figs. S1 to S8 Table S1 Legends for tables S2 to S4 [file sciadv.aea4727_sm.pdf]

Science Advances
AAAS

Supplementary Materials for

Single-cell transcriptomic analysis reveals tumor-immune determinants of lymph node colonization and progression in thyroid cancer

Anthony T. Nguyen *et al.*

Corresponding author: Anthony T. Nguyen, anthony.nguyen@cshs.org; Stephen L. Shiao, stephen.shiao@cshs.org; Allen S. Ho, allen.ho@cshs.org

Sci. Adv. **12**, eaea4727 (2026)
DOI: 10.1126/sciadv.aea4727

The PDF file includes:

Figs. S1 to S8
Table S1
Legends for tables S2 to S4

Other Supplementary Material for this manuscript includes the following:

Tables S2 to S4

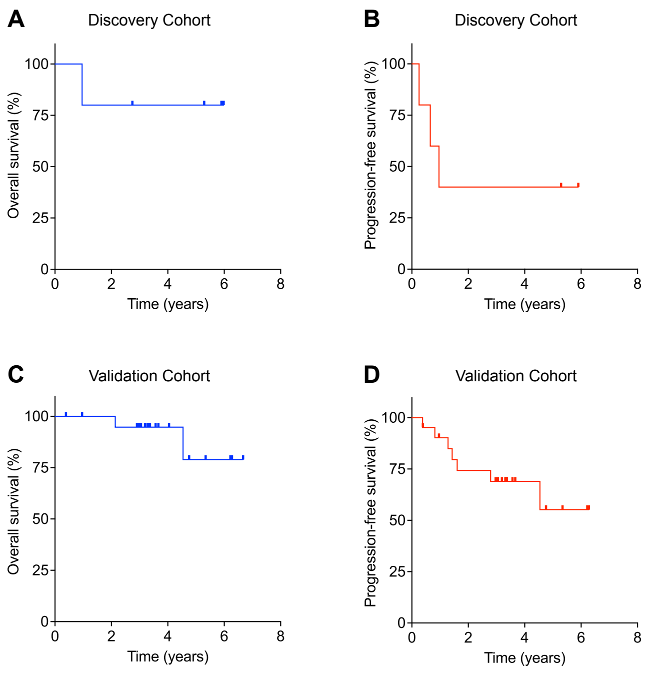

Fig. S1. Oncologic outcomes for the discovery and validation cohorts of thyroid carcinomas. (**A**) Kaplan-Meier estimate for overall survival of the discovery cohort of thyroid carcinoma patients used for single-cell RNA sequencing (scRNA-seq). n = 5 patients. (**B**) Kaplan-Meier plot for progression-free survival for the scRNA-seq discovery cohort of thyroid carcinoma patients. n = 5 patients. (**C**) Kaplan-Meier curve for overall survival of the validation cohort of patients with papillary thyroid carcinoma. n = 21 patients. (**D**) Kaplan-Meier estimate for progression-free survival for patients with papillary thyroid carcinoma in the validation cohort. n = 21 patients.

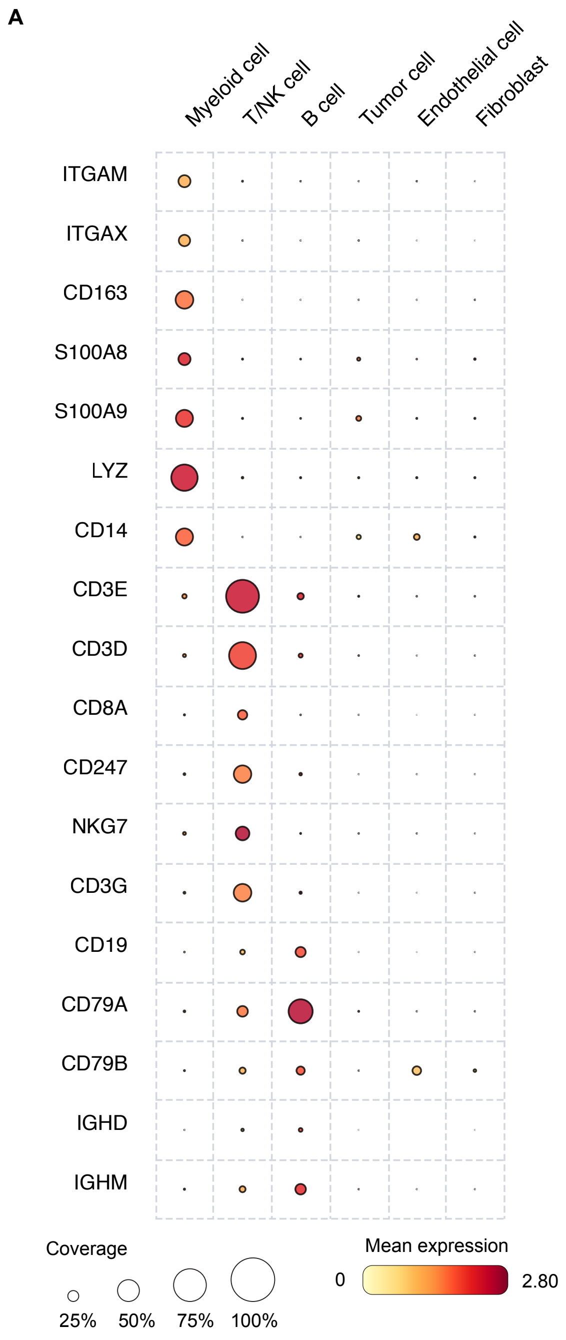

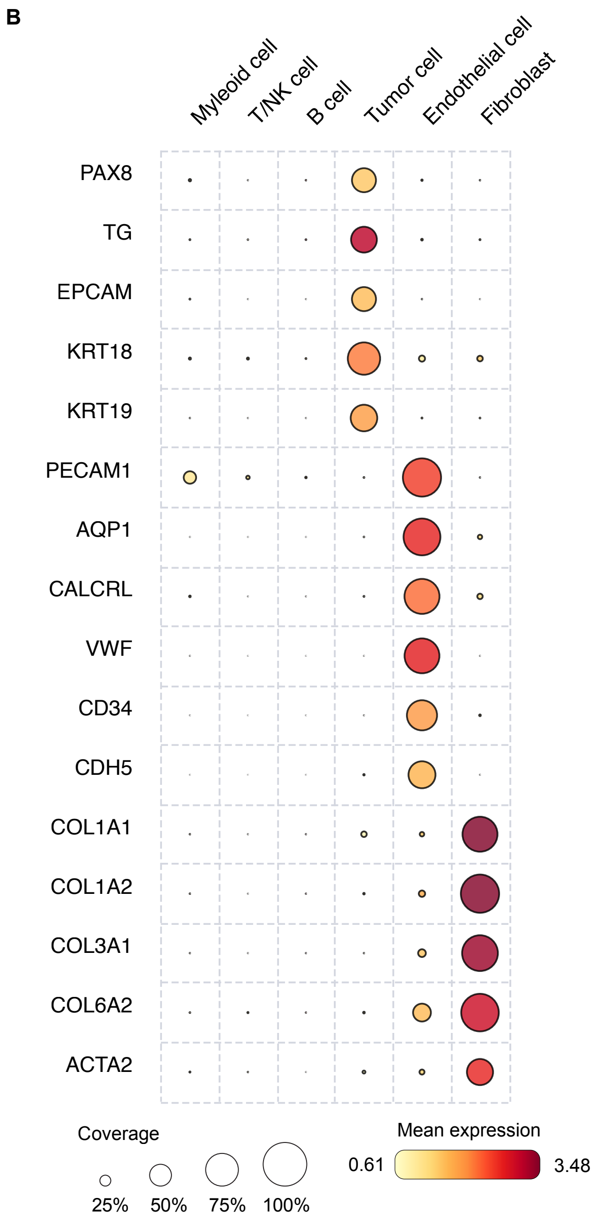

Fig. S2. Expression profiling of 31,026 cells from thyroid carcinoma primary tumors and metastatic lymph nodes. (**A**) Bubble heatmap showing expression of cell type-specific marker genes for tumor-infiltrating leukocytes in the discovery cohort single-cell dataset, as captured with single-cell RNA sequencing. (**B**) Bubble heatmap showing expression of cell type-specific marker genes for non-immune cells in the discovery cohort single-cell dataset. N = 5 pooled patients including primary tumor and metastatic lymph node samples.

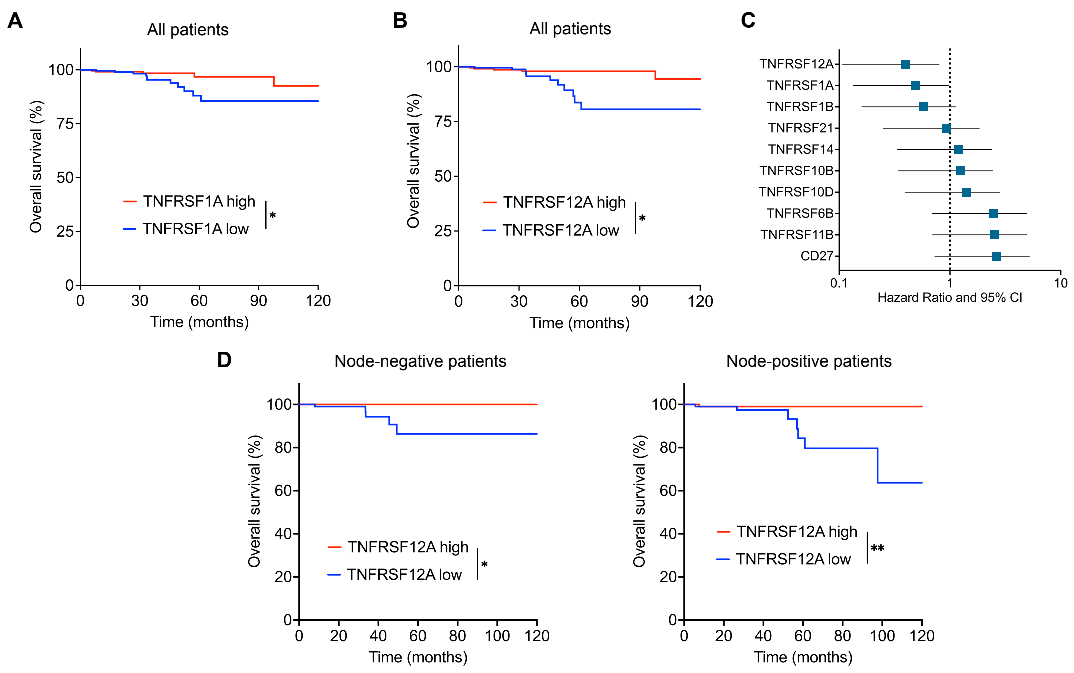

Fig. S3. Association of TNF receptor signaling and survival in thyroid carcinoma. (**A**) Kaplan-Meier plot of overall survival stratifying patients with thyroid carcinoma by expression of *TNFRSF1A*. n = 498 patients. $P = 0.0477$ by log-rank test. (**B**) Kaplan-Meier curve for overall survival in patients with thyroid carcinoma as stratified by high or low expression of *TNFRSF12A*. n = 400 patients. $P = 0.0313$ by log-rank test. (**C**) Forest plot of hazard ratio and 95% confidence interval (CI) for overall survival stratified by expression of TNF receptor superfamily member. (**D**) Kaplan-Meier estimate of overall survival for node-negative patients (left) and node-positive patients (right) stratifying patients by expression of *TNFRSF12A*. n = 226 node-negative patients, n = 223 node-positive patients. $P = 0.014$ for node-negative patients and $P = 0.009$ for node-positive patients by log-rank test. *$P<0.05$, **$P<0.01$.

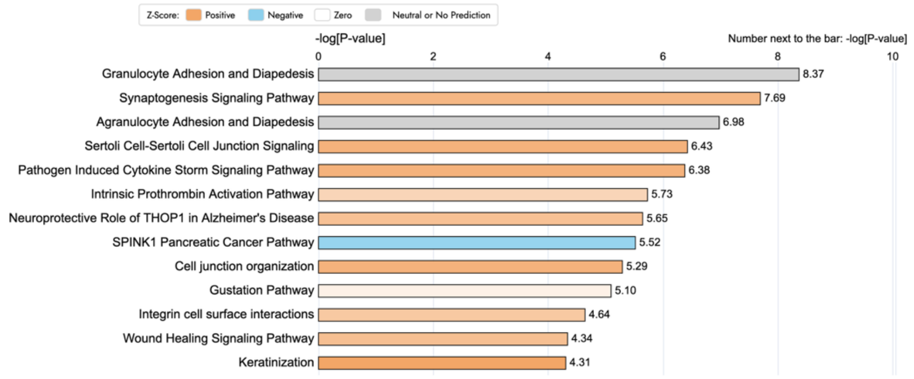

©2000 - 2025 QIAGEN. All rights reserved.

Fig. S4. Significantly activated or inhibited pathways in *TNFRSF12A*-high and -low thyroid tumors by Ingenuity Pathway Analysis (IPA) (**A**) Bar chart showing a summary of canonical pathways identified by IPA (QIAGEN) between *TNFRSF12A*-high and -low thyroid tumors from TCGA. Differentially expressed genes were selected using a $\log_2$ fold-change > 1.5 or < -1.5 and a false discovery rate (FDR) < 0.05. Pathways with potential positive activation are highlighted in orange and pathways with potential negative inhibition are highlighted in blue. The top 13 significant pathways are shown.

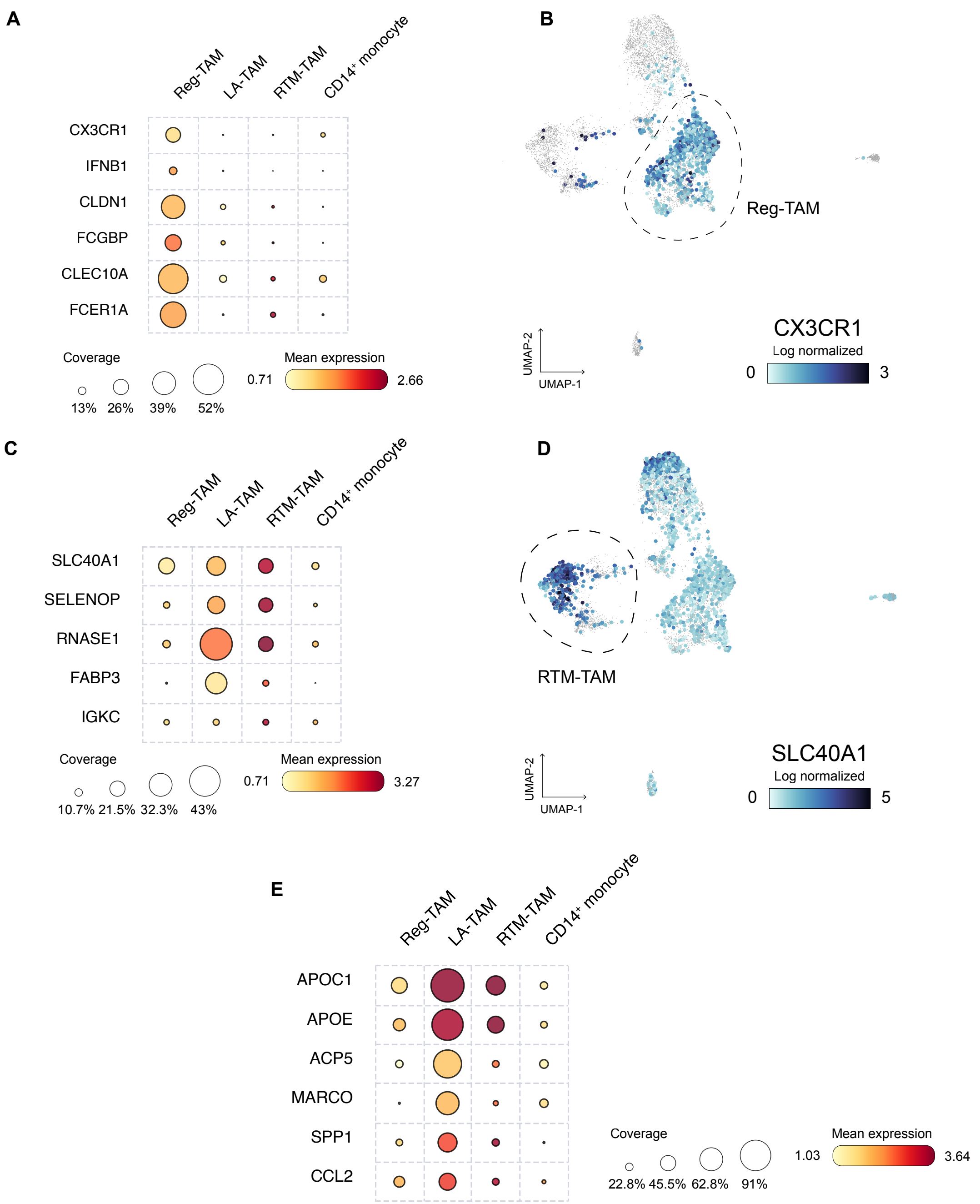

Fig. S5. Diversity of tumor-associated macrophages (TAMs) in thyroid carcinomas by scRNA-seq. (**A**) Expression of cell type-specific marker genes for regulatory TAMs (Reg-TAMs) in the discovery cohort single-cell dataset, as captured with single-cell RNA sequencing. (**B**) UMAP plot showing selective upregulation of CX3CR1 within the Reg-TAM cluster. (**C**) Bubble heatmap showing expression of cell type-specific marker genes for resident-tissue TAMs (RTM-TAMs) in the single-cell dataset. (**D**) UMAP plot showing increased expression of SLC40A1 in the RTM-TAM cluster. (**E**) Bubble heatmap showing expression of cell type-specific marker genes for lipid-associated TAMs (LA-TAMs). N = 5 pooled patients including primary tumor and metastatic lymph node samples.

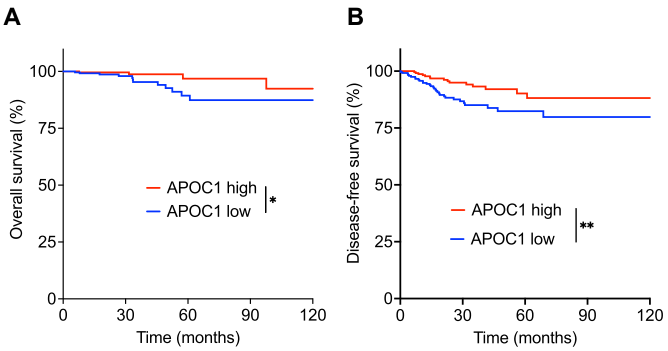

Fig. S6. APOC1, a marker of lipid-associated tumor-associated macrophages is prognostic for survival in thyroid carcinoma. (**A**) Kaplan-Meier plot of overall survival stratifying patients with thyroid carcinoma by expression of *APOC1*. n = 500 patients. $P = 0.043$ by log-rank test. (**B**) Kaplan-Meier estimate of disease-free survival for patients with thyroid carcinoma as stratified by expression of *APOC1*. n = 486 patients. $P = 0.009$ by log-rank test. $^{*}P<0.05$, $^{**}P<0.01$.

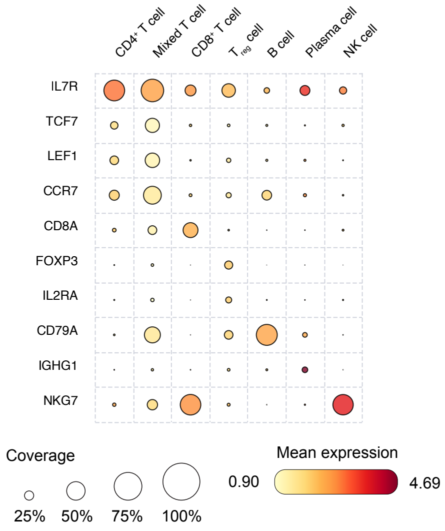

Fig. S7. Subcluster analysis of tumor-infiltrating lymphocytes in thyroid carcinoma. Bubble heatmap showing expression of cell type-specific marker genes for tumor-infiltrating lymphocytes (e.g., T cells, B cells, NK cells) in the discovery cohort single-cell dataset, as captured with single-cell RNA sequencing. Identified cell subtypes include CD4+ T cells (*IL7R*), B cells (*CD79A*), CD8+ T cells (*CD8A*), NK cells (*NKG7*), plasma cells (*IGHG1*), T regulatory cells (*IL2RA*), and mixed T cells (*TCF7*). N = 5 pooled patients including primary tumor and metastatic lymph node samples.

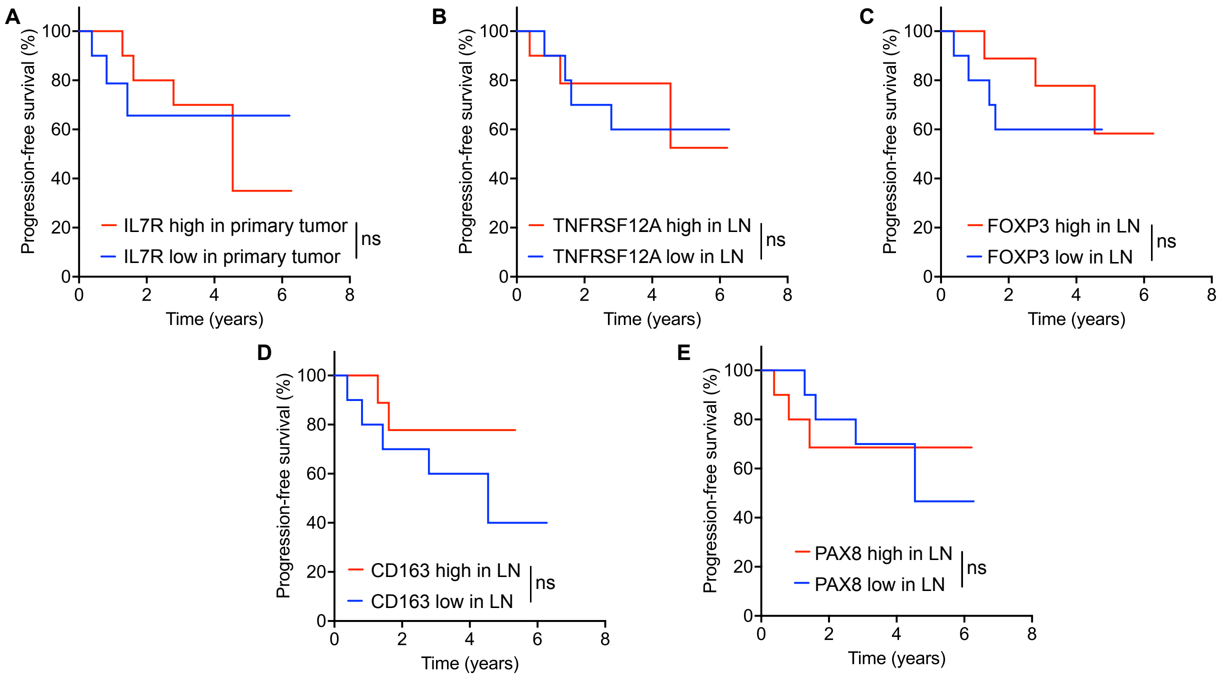

Fig. S8. Other immune targets are not prognostic in thyroid carcinoma. (**A**) Kaplan-Meier plot of progression-free survival stratifying papillary thyroid carcinoma (PTC) patients by primary tumor expression of *IL7R*. n = 20 patients. $P = 0.98$ by log-rank test. (**B**) Kaplan-Meier estimate of progression-survival for PTC patients stratified by lymph node expression of *TNFRSF12A*. n = 21 patients. $P = 0.75$ by log-rank test. (**C**) Kaplan-Meier estimate of progression-survival for PTC patients stratified by lymph node expression of *FOXP3*. n = 21 patients. $P = 0.44$ by log-rank test. (**D**) Kaplan-Meier estimate of progression-survival for PTC patients stratified by lymph node expression of *CD163*. n = 21 patients. $P = 0.25$ by log-rank test. (**E**) Kaplan-Meier estimate of progression-survival for PTC patients stratified by lymph node expression of *PAX8*. n = 21 patients. $P = 0.98$ by log-rank test. LN, lymph node; ns, not significant.

Table S1. Clinicopathologic information for the discovery cohort of patients with thyroid carcinoma for scRNA-seq. Baseline characteristics in patients undergoing total thyroidectomy and lymph node dissection for immune tumor microenvironment profiling. No., number; SD, standard deviation; RAI, radioactive iodine; PTC, papillary thyroid carcinoma. Baseline characteristics were compared using Welch's *t* test for continuous covariates and the Fisher's exact test for categorical covariates.

	Patients, No. (%)		
**Characteristic**	**Discovery (n = 5)**	**Validation (n = 21)**	***P* value**
Age, mean (SD), y	42.9 (17.4)	42.7 (21.0)	0.60
Sex			
Male	1 (20%)	11 (52%)	0.33
Female	4 (80%)	10 (48%)	
Race			
White	2 (40%)	16 (76%)	0.046
Asian	3 (60%)	2 (10%)	
Other	0 (0%)	3 (14%)	
Pathologic T classification			
T1	2 (40%)	10 (48%)	0.19
T2	0 (0%)	4 (19%)	
T3	1 (20%)	6 (29%)	
T4	2 (40%)	1 (5%)	
Pathologic N classification			
N0	0 (0%)	0 (0%)	0.56
N1a	0 (0%)	4 (19%)	
N1b	5 (100%)	17 (81%)	
Size, mean (SD), cm	2.9 (1.4)	2.8 (1.8)	0.90
Margins			
Positive	4 (80%)	9 (43%)	0.32
Negative	1 (20%)	12 (57%)	
Lymphatic invasion			
Yes	3 (60%)	3 (14%)	0.06
No	2 (40%)	18 (86%)	
Extrathyroidal extension			
Yes	2 (40%)	3 (14%)	0.24
No	3 (60%)	18 (86%)	
Examined lymph nodes, mean (SD), number	67 (12)	42 (27)	0.01
Positive lymph nodes, mean (SD), number	18 (15)	19 (17)	0.86
Postoperative RAI			
Yes	5 (100%)	19 (90%)	1.00
No	0 (0%)	2 (10%)	
Histology			
PTC, classical	2 (40%)	17 (81%)	0.13
PTC, tall cell variant	1 (20%)	2 (10%)	
PTC, diffuse sclerosing	1 (20%)	1 (5%)	
PTC, poorly differentiated	0 (0%)	1 (5%)	
Anaplastic	1 (20%)	0 (0%)	

Table S2. Differentially expressed genes identified from TNFRSF12A-high and -low thyroid carcinomas. List of differentially expressed genes (DEGs) comparing TNFRSF12A-high (group A) and TNFRSF12A-low (group B) thyroid carcinomas from The Cancer Genome Atlas. DEGs were identified using a false discovery rate (FDR) < 0.05 and a $\log_2$ fold-change (FC) of >1.5 or < -1.5. *P* values were calculated by Student's t-test. *Q* values or FDR were derived from Benjamini-Hochberg procedure. The table is provided as a separate file.

Table S3. Differentially expressed genes identified from myeloid cells from the primary tumor versus the metastatic lymph node. List of differentially expressed genes (DEGs) comparing myeloid cells from the primary tumor (group 1) versus myeloid cells from the metastatic lymph node (group 2). DEGs were identified using a false discovery rate (FDR) < 0.05 and a $\log_2$ fold-change (FC) of >1 or < -1. The table is provided as a separate file.

Table S4. Differentially expressed genes identified from tumor-infiltrating lymphocytes from the primary tumor versus the metastatic lymph node. List of differentially expressed genes (DEGs) comparing tumor-infiltrating lymphocytes (TILs) from the primary tumor (group 1) versus TILs from the metastatic lymph node (group 2). DEGs were identified using a false discovery rate (FDR) < 0.05 and a $\log_2$ fold-change (FC) of >0.5 or < -0.5. The table is provided as a separate file.
